# Increased child‐evoked activation in the precuneus during facial affect recognition in mothers

**DOI:** 10.1002/hbm.25825

**Published:** 2022-03-12

**Authors:** Irene Sophia Plank, Catherine Hindi Attar, Stefanie Lydia Kunas, Felix Bermpohl, Isabel Dziobek

**Affiliations:** ^1^ Department of Psychology, Faculty of Life Sciences Humboldt‐Universität zu Berlin Berlin Germany; ^2^ Faculty of Philosophy, Berlin School of Mind and Brain Humboldt‐Universität zu Berlin Berlin Germany; ^3^ Department of Psychiatry and Neurosciences CCM Charité – Universitätsmedizin Berlin, Corporate Member of Freie Universität Berlin and Humboldt‐Universität zu Berlin Berlin Germany; ^4^ Einstein Center for Neurosciences Charité – Universitätsmedizin Berlin, Corporate Member of Freie Universität Berlin and Humboldt‐Universität zu Berlin Berlin Germany

**Keywords:** affect recognition, child‐evoked activation, children, emotion, facial affect recognition, fMRI, motherhood

## Abstract

Successful parenting requires constant inferring of affective states. Especially vital is the correct identification of facial affect. Previous studies have shown that infant faces are processed preferentially compared to adult faces both on the behavioural and the neural level. This study specifically investigates the child‐evoked neural responses to affective faces and their modulation by motherhood and attention to affect. To do so, we used a paradigm to measure neural responses during both explicit and implicit facial affect recognition (FAR) in mothers and non‐mothers using child and adult faces. Increased activation to child compared to adult faces was found for mothers and non‐mothers in face processing areas (bilateral fusiform gyri) and areas associated with social understanding (bilateral insulae and medial superior frontal gyrus) when pooling implicit and explicit affect recognition. Furthermore, this child‐evoked activation was modulated by motherhood with an increase in mothers compared to non‐mothers in the left precuneus. Additionally, explicitly recognising the affect increased child‐evoked activation in the medial superior frontal gyrus in both mothers and non‐mothers. These results suggest preferential treatment of affective child over adult faces, modulated by motherhood and attention to affect.

## Graphical abstract

Increased activation to child compared to adult faces was found for mothers and non‐mothers in face processing areas (bilateral fusiform gyri) and areas associated with social understanding (bilateral insulae and medial superior frontal gyrus) during implicit and explicit affect recognition. Furthermore, this child‐evoked activation was modulated by motherhood with an increase in mothers compared to non‐mothers in the left precuneus. Additionally, explicitly recognising the affect increased child‐evoked activation in the medial superior frontal gyrus in both mothers and non‐mothers.

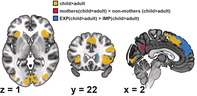

## 1 INTRODUCTION

For a species to succeed, its offspring must be successfully raised to adulthood. It is, therefore, not surprising that infants and children are treated differently than adults (Kringelbach, Stark, Alexander, Bornstein, & Stein, 2017; Lorenz, 1943). They receive more attention and are more rewarding, especially for parents (Lucion et al., 2017; Luo, Li, & Lee, 2011; Thompson‐Booth et al., 2014a, 2014b). This modulation by parenthood is not surprising given that parents need to constantly infer their offspring's thoughts and emotions as well as react to them (Pereira & Ferreira, 2016). These processes are referred to as social understanding. Social understanding has been shown to be modulated by the target (e.g., infant vs. adult, Proverbio, Brignone, Matarazzo, Del Zotto, & Zani, 2006) and the perceiver (e.g., mother vs. non‐mother, Plank, Hindi Attar, et al., 2021a; Plank, Hindi Attar, et al., 2021b).

**FIGURE 1:**
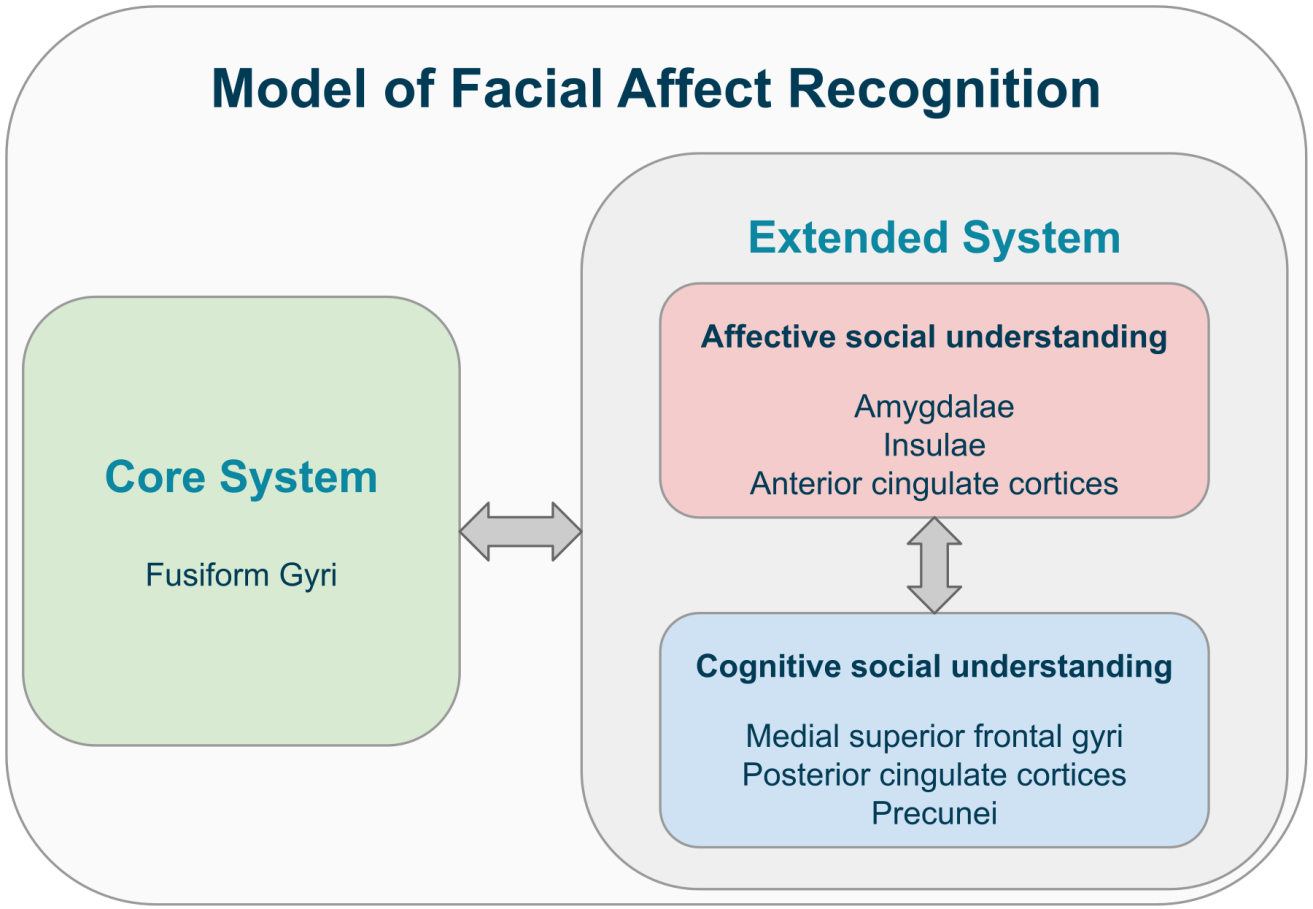
Box model showing the conceptualisation of recognising facial affect and associated brain areas. The core system focuses on the visual processing of the faces, especially in the fusiform gyri. The extended system is associated with the advanced processing of facial stimuli. Here, two aspects of social understanding go hand in hand: affective social understanding focusing on understanding by creating affective states and cognitive social understanding focusing on abstract inferences based on the available information

One of the most important modes of non‐verbal communication is facial expressions. Recognising the emotion of a face is a vital process in many human interactions. Facial affect recognition (FAR) combines several processes: one needs to process the visual information, extract relevant features connected to the portrayed information and infer the affective state based on these features. Additionally, affective faces can also induce an emotional reaction in the perceiver. Therefore, FAR combines affective and cognitive aspects of social understanding (Kanske, 2018; Shamay‐Tsoory, 2011). In Gobbini and Haxby's model of *face recognition*, a core system of processing visual appearance is complemented by an extended system that they refer to as person knowledge and emotion, but which can also be conceptualised as cognitive and affective social understanding, respectively (Gobbini & Haxby, 2007). We have adapted this model by integrating findings on social understanding generally and affect recognition specifically to construct a model of *facial affect recognition* (Kogler, Müller, Werminghausen, Eickhoff, & Derntl, 2020; Schirmer & Adolphs, 2017). The resulting model, shown in Figure 1, focuses on three main processes, which interact with each other: face processing as the core system in the fusiform gyri, affective social understanding in the amygdalae, insulae and anterior cingulate cortices as well as cognitive social understanding of emotions in the medial superior frontal gyri, posterior cingulate cortices and the precunei. These areas have been implicated in the so‐called ‘parental brain’ (Feldman, 2015), making them important regions of interest (ROIs) when investigating potential modulatory effects of parenthood.

When parents encounter the face of their own child as opposed to the face of an unfamiliar child, they show increased neural responses in areas of the extended system, including the insula, amygdala, anterior cingulate cortex and precuneus (Kluczniok et al., 2017; Leibenluft, Gobbini, Harrison, & Haxby, 2004). While own children are preferentially treated compared to unfamiliar children, the unfamiliar child faces also lead to increased activation compared to adult faces in mothers in the fusiform gyrus of the core system as well as areas of the extended system, including the insula and the precuneus (Leibenluft et al., 2004). Notably, the insula is also associated with pleasure and reward (Berridge & Kringelbach, 2015; Vestergaard & Schultz, 2020) as well as emotional salience processing (Phan et al., 2004). Similarly, a study on nulliparous women showed increased activation in the core system in response to unfamiliar affective infant compared to adult faces (Li et al., 2016). This suggests that preferential treatment of child and infant faces compared to adult faces applies to both mothers and non‐mothers. So far, however, no study has investigated child‐evoked activation in both mothers and non‐mothers in response to affective child versus adult faces. Child‐evoked activation could play a role in the rewarding and motivating effects of infant faces observed on the behavioural level (Lucion et al., 2017; Luo et al., 2011; Thompson‐Booth et al., 2014a, 2014b).

Studies investigating modulations of this child‐evoked activation are still scarce. Nishitani, Doi, Koyama, and Shinohara (2011) were the first to show a modulation of child‐evoked activation during FAR by motherhood using functional near‐infrared spectroscopy (fNIRS). They reported increased activation in the left prefrontal cortex in mothers compared to non‐mothers, but only when processing infant and not adult faces, suggesting that child‐evoked activation is modulated by motherhood (Nishitani et al., 2011). A study using affective infant faces found increased neural activity in mothers compared to non‐mothers in the core system (bilateral fusiform gyri) and bilateral frontal areas (Zhang et al., 2020). However, the authors did not use adult stimuli as a comparison allowing only tentative conclusions on the modulation of child‐evoked activation by motherhood. It is, therefore, an open question, which areas associated with child‐evoked activation are modulated by motherhood.

Additionally, it is still unclear whether directing the attention to the displayed emotion in an explicit as opposed to away from it in an implicit affect recognition task modulates child‐evoked activation. Studies comparing implicit and explicit affect recognition have shown increased activation in areas of affective social understanding during explicit affect recognition (Gur et al., 2002; Habel et al., 2007; but see also Critchley et al., 2000). Since children's preferential treatment seems partly due to an attentional bias, child‐evoked activation may be increased in the explicit affect recognition task (Lucion et al., 2017). It is also possible that the modulation of child‐evoked activation by motherhood is caused by an increased attentional bias for infant and child faces in mothers compared to non‐mothers (Thompson‐Booth et al., 2014b). This suggests the possibility that the modulation by motherhood is decreased when attention (also of non‐mothers) is directed to the displayed emotion, resulting in less difference between mothers and non‐mothers in an explicit compared to an implicit affect recognition task.

This functional magnetic resonance imaging (fMRI) study aimed to investigate neural responses to affective child versus adult faces, henceforth referred to as child‐evoked activation, and potential modulations of this child‐evoked activation. Specifically, we were interested in the influence of motherhood and attention to affect. We adapted an established paradigm (Mier et al., 2010) to measure neural activation during both explicit and implicit FAR, allowing us to assess whether child‐evoked activation is increased by directing attention to the portrayed emotion. To assess child‐evoked activation, we used both affective child and adult faces in our study and focused on activation that was stronger in response to child compared to adult faces. As we are interested in primary care parents, we decided to focus on mothers since they are more likely to provide primary care for their children than fathers in Germany (BMFSFJ, 2019; Bundeszentrale für politische Bildung, 2021). Based on previous literature and our conceptualisation of FAR (Leibenluft et al., 2004; Li et al., 2016; Zhang et al., 2020), we hypothesised child‐evoked activation in areas of our model of FAR (Hypothesis 1: child > adult). We also expected child‐evoked activation to be increased in mothers compared to non‐mothers (Hypothesis 2: mothers_child>adult_ > non‐mothers_child>adult_). Additionally, we hypothesised stronger child‐evoked activation in the explicit than in the implicit FAR task due to the increased attention to the affective content of the faces (Hypothesis 3: EXP_child>adult_ > IMP_child>adult_). Lastly, we expected the modulation of child‐evoked activation by motherhood to be decreased in the explicit compared to the implicit affect recognition task (Hypothesis 4: interaction motherhood * attention).

## 2 METHODS

The study design, sample size, data collection procedures, behavioural analyses and Hypotheses 1 and 2 have been preregistered prior to data collection (available at https://osf.io/kb675). However, ROIs and neuroimaging analyses as well as all Hypotheses 3 and 4 have not been preregistered.

### 2.1 Participants

Based on an a priori power estimation, we intended to analyse 54 participants (*G* * Power: 2 × 2 mixed ANOVA for within‐between interaction with *f* = 0.25, *α* = .05, *[1* − *β]* = 0.95, corr = .5; Faul, Erdfelder, Lang, & Buchner, 2007). We only included women who were MRI compatible, right‐handed, cisgender, between 25 and 50 years of age, had sufficient knowledge of German and were of good mental and neurological health (assessed during a semi‐structured interview). Three participants had to be excluded due to chance or worse behavioural performance in the task. Two participants had to be excluded, one because of excessive head motion and one during visual inspection. Due to the outbreak of Covid‐19, we could not replace them and analysed 50 participants, of which 26 were non‐mothers (mean age 35.92 years) and 24 were mothers (mean age 38.38 years). Our groups were of comparable age, intelligence and socioeconomic status (see Table 1). Mothers were the primary caretaker of at least one biological child in the age range of the child stimuli (4–10 years of age). We excluded non‐mothers if they spent significant time with children (private or professional). This study was positively assessed by the Ethics committee of the Charité—Universitätsmedizin Berlin and conducted in accordance with the Declaration of Helsinki. All women were informed about the study before giving their written consent. They received monetary compensation for their participation in the study.

**TABLE 1:** Comparison of mothers and non‐mothers using Bayesian Mann–Whitney‐*U* tests and a Bayesian contingency table

Measurement	Mothers	Non‐mothers	*BF*_10_	*W*
Age	38.4 ± 0.8	35.9 ± 1.4	0.130	251.00
Number of children	2.04 ± 0.17 (max. 4)	—	—	—
Duration of motherhood	9.63 ± 0.94 (max. 22)	—	—	—
ECR‐rs	25.1 ± 1.7	31.8 ± 1.7	0.770	446.00
ERQ	40.7 ± 1.3	42.9 ± 1.4	0.117	377.00
Importance of having children (0 to 4)	3.7 ± 0.1	2.7 ± 0.3	0.366	203.50
IRI‐emp	44.6 ± 1.2	42.7 ± 1.4	0.130	269.50
IRI‐PT	15.1 ± 0.4	15.0 ± 0.4	0.081	315.50
KSE‐G	1.8 ± 0.1	2.0 ± 0.1	0.188	392.50
MinIQ	28.2 ± 2.3	30.6 ± 2.2	0.089	334.00
Mood state (0–4)	3.0 ± 0.1	3.0 ± 0.1	0.081	311.00
Single (proportion of group)	25%	73%	108	—
SES (3–21)	14.1 ± 0.7	15.4 ± 0.8	0.141	187.50
TAS	38.7 ± 1.5	40.0 ± 1.9	0.081	329.00

*Note*: Columns show averages, standard errors, corrected Bayes factor and *W* for each test. All comparisons indicate no differences between groups. This table has been reproduced from Plank, Hindi Attar, Kunas, Dziobek, and Bermpohl (2021b).

Abbreviations: ECR‐RS, experiences in close relationships—relationship structures; ERQ, emotion regulation questionnaire; IRI‐emp, interpersonal reactivity index, empathy score; IRI‐PT, interpersonal reactivity index, subscale perspective taking; KSE, Kurzskala Soziale Erwünschtheit (short scale social desirability), positive and negative subscale; SES, socio‐economic status; TAS, Toronto alexithymia scale.

### 2.2 Facial affect recognition task

We adapted our task from Mier et al. (2010). Participants were asked to match affective (angry, happy and afraid) adult and child faces to sentences describing the depicted emotion or an unrelated physical feature. When asked to match a sentence describing the facial affect, participants had to perform explicit FAR, while assessing only the physical features led to an implicit recognition of the affect. In the following, we will refer to the explicit FAR condition as EXP and the implicit FAR task as IMP. The following sentences were used in the EXP task (translated from German): ‘This child/person is angry’, ‘This child/person is afraid’ and ‘This child/person is happy’. In the IMP task, the following sentences were used: ‘This child/person is older than 8/25 years’, ‘This child/person is taller than 130/175 cm’ and ‘This child/person is heavier than 25/70 kg’. Additional sentences described action intentions, but these are not the subject of this article. Associated results have been published in Plank, Hindi Attar, Kunas, Dziobek, and Bermpohl (2021b). The faces of 24 identities were taken from established databases (Dartmouth Database of Children's Faces, Dalrymple, Gomez, & Duchaine, 2013; Developmental Emotional Faces Stimulus Set, Meuwissen, Anderson, & Zelazo, 2017; NimStim set of facial expressions, Tottenham et al., 2009). Identities were balanced over age group (adult and child) and gender (female and male). To increase task difficulty, all facial expressions were shown at 70% intensity of anger, fear and happiness, respectively. Each identity was shown displaying each emotion once per task. All faces were presented in greyscale.

**FIGURE 2:**
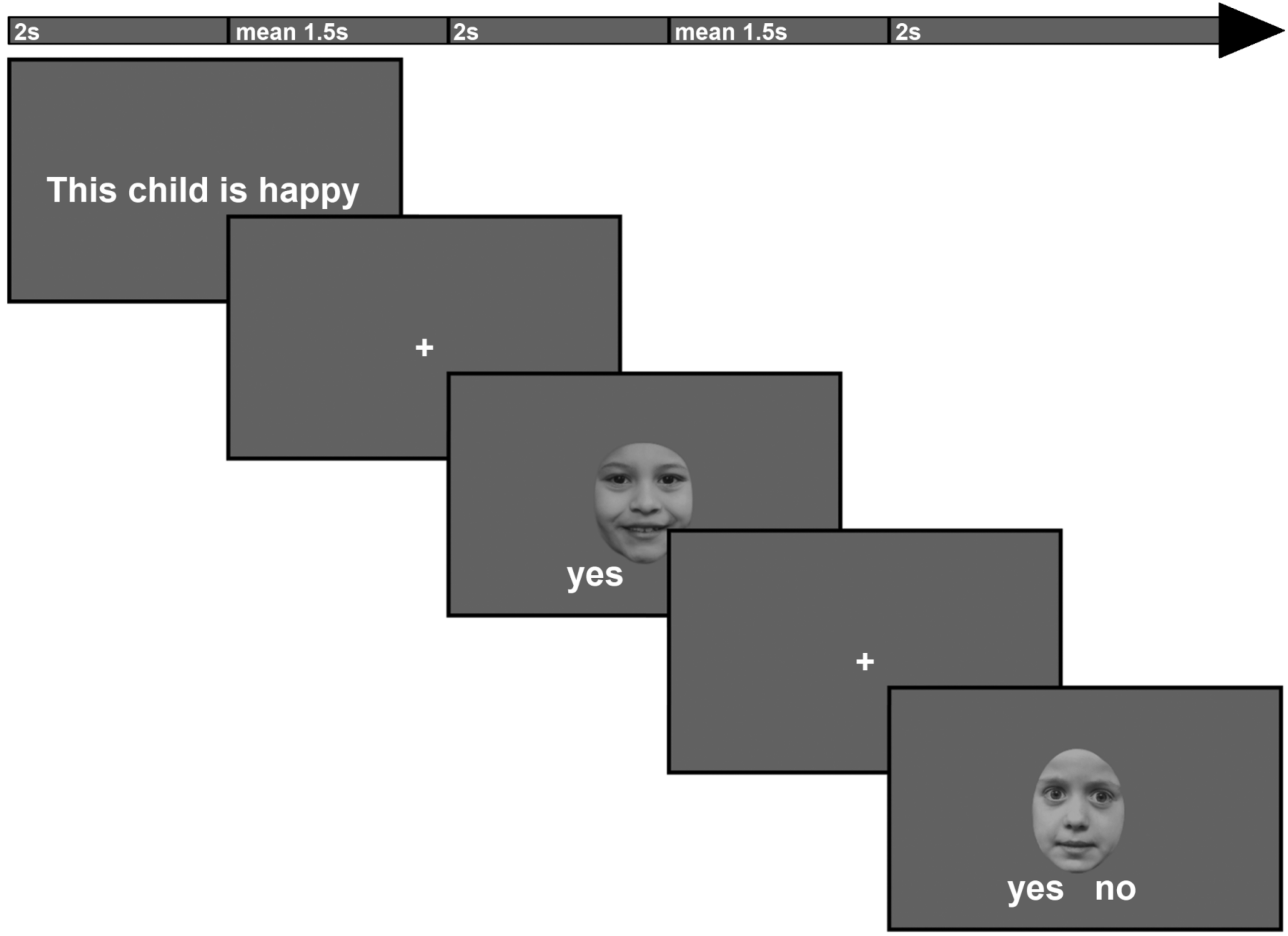
Schema of the beginning of a block in the EXP condition. In each block, one sentence is followed by four faces, with participants having to decide for each face whether it fits the sentence or not. Sentences and faces are presented for 2 s each. Before each face, a fixation cross is presented for 1.5 s on average

The FAR task was presented in three runs of 36 blocks each. Each block started with a sentence of either the IMP or the EXP condition, followed by four trials of either adult or child faces (see Figure 2). Similarly to previous studies by Mier and colleagues (Mier et al., 2010, 2013; Yan et al., 2020), the task was designed to examine general FAR irrespective of the specific emotion, therefore, one block contained all three emotions. Two of the four trials in each block matched the introducing sentence, while the others did not. The order of matching and mismatching trials was randomised. The task and protagonist of the trials in one block are consistent, resulting in 72 stimuli per task (EXP and IMP), 36 of which were adult and 36 of which were child stimuli. We pseudo‐randomised the order of the blocks so that a maximum of two subsequent blocks were of the same condition. Both sentences and faces were displayed for 2 s each. Before the presentation of each face, a fixation cross is presented for 1.5 s on average (based on a truncated exponential, *λ* = 0.5565, *min* = 1, *max* = 3) and a grey blank screen was presented between blocks for 9.5 s on average (based on a uniform distribution, *min* = 8, *max* = 11).

### 2.3 Experimental procedure

After we informed participants about the study and scanning procedures, they had the opportunity to ask questions before signing the consent form. Following this, we conducted a semi‐structured interview on socio‐demographics and family status with them. Based on their answers, we computed a score measuring their socio‐economic status (SES, including education, career and net equivalent income; Lampert, Kroll, Müters, & Stolzenberg, 2013). Next, they completed the MinIQ, a short screening to estimate their IQ (Baudson & Preckel, 2016). Then, they filled out questionnaires to measure emotion regulation (ERQ; Abler & Kessler, 2009), relationship attachment (ECR‐RS; Fraley, Heffernan, Vicary, & Brumbaugh, 2011), social desirability (KSE‐G; Kemper, Beierlein, Bensch, Kovaleva, & Rammstedt, 2012), alexithymia (TAS‐20; Popp et al., 2008) as well as trait empathy and perspective‐taking (both IRI; Paulus, 2009). Last, they performed two tasks in the scanner, an empathy for pain task (Plank, Hindi Attar, Kunas, Dziobek, & Bermpohl, 2021a) and the here presented task of which the ToM condition has been published separately (Plank, Hindi Attar, Kunas, Dziobek, & Bermpohl, 2021b). In the empathy for pain task, participants saw children and adults in painful or non‐painful situations showing body parts. The situations were introduced with faces, none of which were used in this FAR task. The empathy for pain task was always presented before the FAR task with a short break in between to decrease influences. Additionally, resting‐state data were collected. In total, the experiment took about 90 min, of which 60 min were spent in the fMRI scanner.

### 2.4 Sample characteristics and task performance

All behavioural analyses were performed in JASP (JASP Team, 2020) and interpreted based on the adaptation of Jeffrey's scheme used in JASP (Goss‐Sampson, 2020). We computed Bayesian Mann–Whitney‐*U* tests based on 10,000 random samples and corrected for multiple comparisons with Westfall's method (de Jong, 2019; Westfall, Johnson, & Utts, 1997) for all covariates (using 10,000 random samples) except for relationship status where we used a Bayesian contingency table with independent multinomial sampling. We analysed response times to evaluate the behavioural performance in EXP and IMP. This deviates from the preregistered score integrating accuracy and response time because in the IMP condition we added sentences where we did not know the correct answer resulting in no associated accuracies. We entered the response times into a Bayesian mixed ANOVA with predictors motherhood, task (EXP or IMP) and protagonist (child or adult). We intended to add variables where the Mann–Whitney‐*U* tests indicated group differences to the ANOVA as covariates, however, none fulfilled this condition.

### 2.5 fMRI data acquisition

All neuroimaging data were collected at the Berlin Center for Advanced Neuroimaging using a 3T scanner (Siemens Magnetom Prisma, Siemens Medical Solutions, Erlangen, Germany). We acquired structural images with a T1‐weighted magnetically prepared rapid acquisition gradient echo (176 slices; voxel size = 1 mm^3^; TR = 2,539 ms; FA = 7°; FOV = 256 mm). Next, we collected field maps (32 slices à 3 mm; TR = 400 ms; TE_1_ = 5.19 ms; TE_2_ = 7.65 ms; FA = 60°; FOV = 192 mm) which were followed by T2*‐weighted echo‐planar imaging (EPI). The EPI sequence measuring brain activation during the FAR task consisted of three runs (244 scans each, 32 slices, voxel size = 3 mm^3^; TR = 2,000 s; TE = 30 ms; FA = 78°; FOV = 192 mm).

### 2.6 fMRI data pre‐processing

We performed pre‐processing with *fMRIPrep* 20.0.6 (Esteban et al., 2019). For details on the pre‐processing, see the automatically generated description provided by *fMRIPrep* in the supplementary materials. We corrected the anatomical scans for intensity non‐uniformity, skull‐stripped and segmented them before using them as T1‐weighted references. For each run, we performed fieldmap correction, coregistration, realignment and slice time correction. All images were normalised to the Montreal Neurological Institute space (MNI152NLin2009cAsym, Fonov, Evans, McKinstry, Almli, & Collins, 2011). We only included participants in the analyses who moved less than 3 mm in any direction (equivalent to one voxel size). After pre‐processing the scans with *fMRIPrep*, we detrended (Macey, Macey, Kumar, & Harper, 2004) and smoothed them using a 6 mm Gaussian kernel in SPM12 (Wellcome Department of Imaging Neuroscience, University College London, UK, 2014).

### 2.7 fMRI analysis

All analysis of the functional neuroimaging data were conducted based on the general linear model (GLM) in SPM12. First, we specified and estimated a GLM for each participant with four covariates of interest (EXP_child_, EXP_adult_, IMP_child_ and IMP_adult_). We also included the runs, conditions of no interest and the response times as covariates of no interest. The response time was added to control for differences in response time between conditions. Since we were interested in child‐evoked activation, we created three differential contrasts for each participant: one measuring child‐evoked activation in the EXP task (EXP_child>adult_), one measuring child‐evoked activation in the IMP task (IMP_child>adult_) and one measuring child‐evoked activation pooled over both tasks (EXP + IMP: child > adult). The pooled differential contrast was used on the second level in a one‐sample *t*‐test over both mothers and non‐mothers to evaluate Hypothesis 1. The task‐specific differential contrasts were entered in a flexible factorial on the second level, including the factors subjects, motherhood (yes or no), attention to affect (EXP and IMP) and the interaction between motherhood and attention. To evaluate Hypothesis 2, we pooled over tasks and compared ‘mother_child>adult_’ with ‘non‐mother_child>adult_’ and for Hypothesis 3, we pooled over groups and compared ‘EXP_child>adult_’ and ‘IMP_child>adult_’. Lastly, we computed the interaction between motherhood and attention to affect in the flexible factorial to evaluate hypothesis 4. We performed a ROI analysis using one single mask to perform a small‐volume correction for all contrasts evaluating our hypotheses. This mask included the following regions (all bilaterally): insulae, amygdalae, fusiform gyri, medial superior frontal gyri, precunei and posterior cingulate cortices. The mask was created using MARINA (Walter et al., 2003) based on AAL (Tzourio‐Mazoyer et al., 2002). We also explored further differences outside the mask using a whole‐brain approach. All results are family‐wise error (FWE) corrected with *p* < .05 on the cluster level. We used a grey matter mask on all whole‐brain contrasts for visual purposes (at least 10% probability for grey matter based on the tissue probability map provided by SPM12).

## 3 RESULTS

### 3.1 Sample characteristics

Generally, the samples of mothers and non‐mothers collected in this study were comparable. Computed Bayesian Mann–Whitney‐*U* tests did not reveal significant differences between mothers and non‐mothers concerning age, socio‐economic status, mood state, intelligence or the importance they place on having children nor in any of the questionnaires (see Table 1). In addition, none of the questionnaires indicated any differences between mothers and non‐mothers. Even so, the Bayesian Contingency table revealed decisive evidence for differences between mothers and non‐mothers in relationship status with mothers less likely to be single (25%) compared to non‐mothers (73%, *BF*_10_ = 108).

### 3.2 Task performance

**FIGURE 3:**
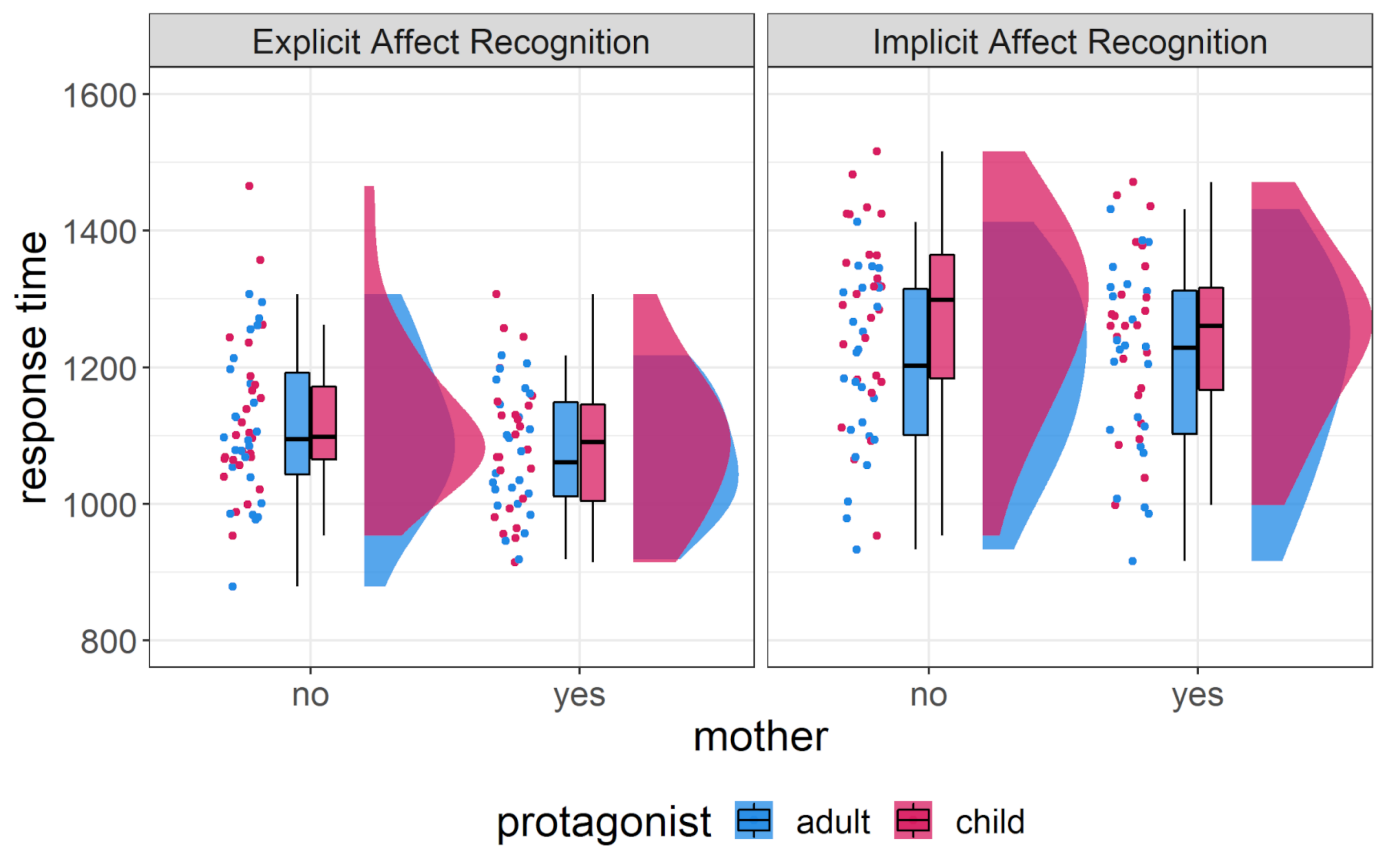
Performance in the task as measured by response times. Each dot represents the average response times of a participant in a condition. Boxplots show median response times, first and third quartiles as hinges as well as whiskers extending 1.5 times the interquartile range for each condition separately. There were no differences between mothers and non‐mothers, but the response time was influenced by task, protagonist and potentially the interaction of task and protagonist. Matching sentences with physical descriptions unrelated to the emotion in the IMP task took longer than matching the portrayed emotion in the EXP task. Additionally, child faces led to longer response times than adult faces

Response times did not differ between mothers and non‐mothers. However, there were differences due to the task, protagonist and the interaction between task and protagonist, with the IMP task leading to longer response times than the EXP task and children leading to longer response times than adults (see Figure 3). Decisive evidence supports the model including these three predictors (*BF*_10_ = 3.19e + 20), making it more than twice as likely as the second‐best model, additionally including the factor motherhood (for all Bayes factors see the Supporting Information). Inclusion Bayes factors across matched models, on the one hand, reveal decisive evidence for the predictor task (*BF*_incl_ = 2.90e + 19), very strong evidence for the protagonist (*BF*_incl_ = 69.74) and anecdotal evidence for the interaction of task and protagonist (*BF*_incl_ = 2.39). On the other hand, evidence was revealed *against* the predictor motherhood (anecdotal: *BF*_incl_ = 0.44), the interaction between task and motherhood (anecdotal: *BF*_incl_ = 0.34), the interaction between protagonist and motherhood (moderate: *BF*_incl_ = 0.28) and the three‐way interaction (anecdotal: *BF*_incl_ = 0.40). This indicates that even though motherhood did not influence response time, task and protagonist as well as their interaction did, which strengthens the decision to include response time in the first level GLM of the neuroimaging analysis.

### 3.3 fMRI results

**FIGURE 4:**
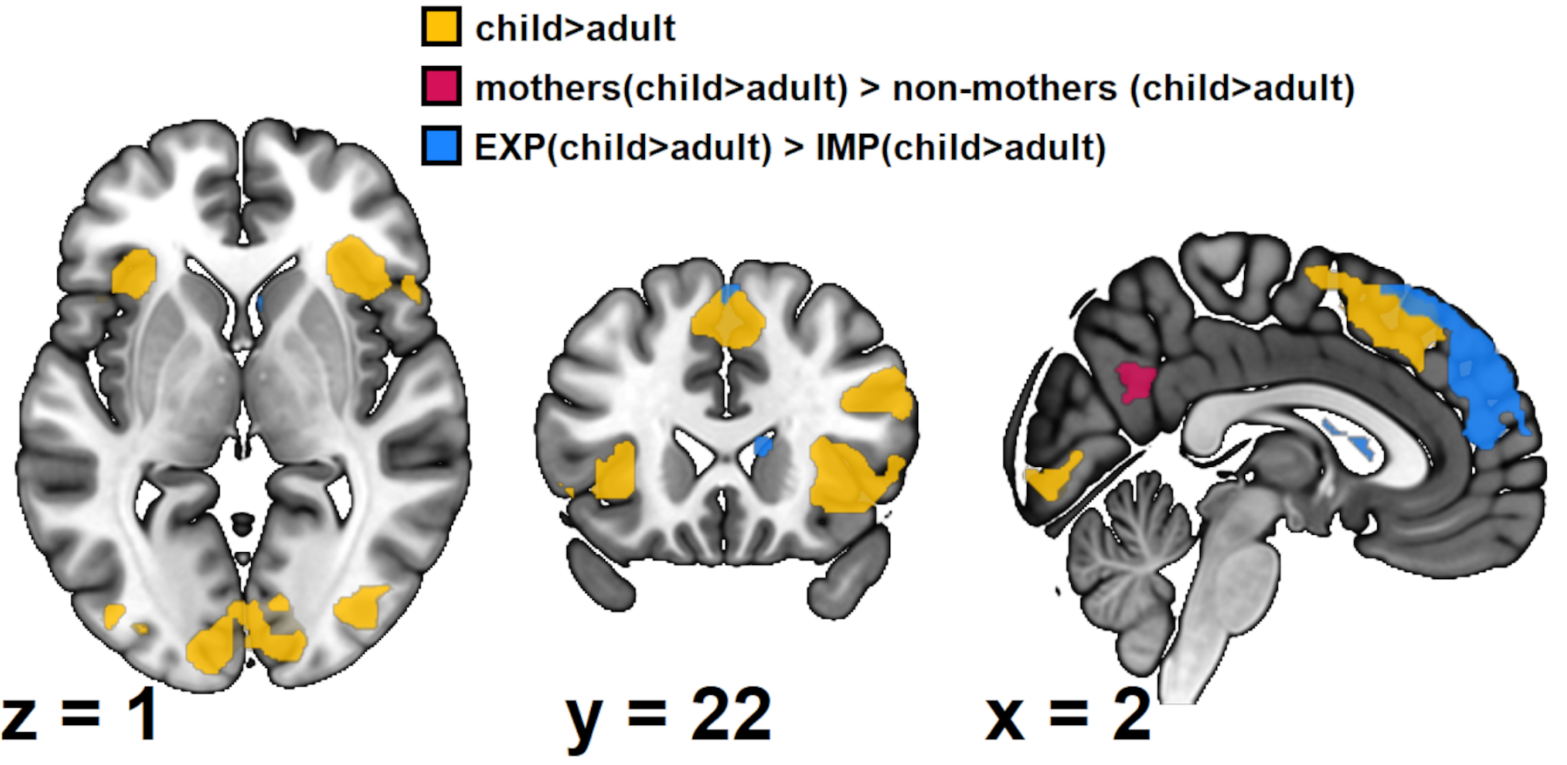
Results of the whole‐brain analyses, FWE‐corrected with *p* < .05 on the cluster‐level. We used a 10% grey‐matter mask for aesthetic purposes and created the image using MRICroGL (Rorden & Brett, 2000)

Both the hypotheses‐guided ROI and the explorative whole‐brain analyses revealed child‐evoked activation in both mothers and non‐mothers, which was further modulated by motherhood and attention to affect (see Figure 4). The hypotheses‐guided ROI analyses focused on the areas of our FAR model, as shown in Figure 1. To investigate Hypothesis 1, we focused on child‐evoked activation in these ROIs. It revealed child‐evoked activation in the right fusiform gyrus (*k*_*E*_ = 76, *T* = 7.15), medial superior frontal gyrus (*k*_*E*_ = 305, *T* = 6.88) as well as the bilateral insulae (right: *k*_*E*_ = 195, *T* = 7.61; left: *k*_*E*_ = 209, *T* = 6.42), therefore supporting hypothesis 1 (child>adult). This child‐evoked activation was significantly stronger in mothers in the left precuneus compared to child‐evoked activation in non‐mothers pooled over both tasks (*k*_*E*_ = 198, *T* = 4.80; mothers_child>adult_ > non‐mothers_child>adult_). This partly supports Hypothesis 2 by showing increased child‐evoked activation in mothers in the extended system. However, there was no difference in neural response in areas associated with affective social understanding. Additionally, child‐evoked activation as a response to the EXP task was stronger than in response to the IMP task in the left medial superior frontal gyrus for mothers and non‐mothers combined (*k*_*E*_ = 1,188, *T* = 9.53), partly supporting Hypothesis 3 (EXP_child>adult_ > IMP_child>adult_). Again, modulations affected an area associated with cognitive social understanding but not areas associated with affective social understanding. There were no significant differences in activation in any of the ROIs in the contrasts ‘non‐mothers_child>adult_ > mothers_child>adult_’, ‘IMP_child>adult_ > EXP_child>adult_’ or in the interaction between child‐evoked activation, motherhood and attention to affect; therefore, Hypothesis 4 was not supported.

The explorative whole‐brain analyses mirror the ROI results (see Table 2). Child‐evoked activation was found in both the core and the extended system (Hypothesis 1). In addition, this child‐evoked activation was increased in the left precuneus in mothers (Hypothesis 2) and the left medial superior frontal gyrus when performing explicit compared to implicit FAR (Hypothesis 3). Again, there were no significant differences in child‐evoked activation in other comparisons (Hypothesis 4).

**TABLE 2:** Results of the explorative whole‐brain analysis

Region	BA	H	Cluster size	*t*‐value	*x*	*y*	*z*
*Child > adult*							
Lingual gyrus inc. fusiform gyrus*	18	R	3,451	8.23	12	−87	−7
Calcarine	18	L		8.02	−13	−95	−3
Lingual gyrus inc. fusiform gyrus*	18	L		7.45	−11	−87	−11
Insula*	13	R	2,442	7.80	34	26	−3
Inferior frontal gyrus	47	R		6.86	42	28	−5
Inferior frontal gyrus	44	R		6.49	44	16	28
Medial superior frontal gyrus*	8	R	954	6.88	2	32	48
Supplementary motor area	8	R		6.23	4	22	50
Supplementary motor area	6	L		5.90	−3	12	62
Inferior frontal gyrus inc. insula *	47	L	352	6.42	−35	26	−1
Inferior frontal gyrus	47	L		4.51	−43	18	−3
Inferior frontal gyrus	45	L		3.92	−53	20	−3
Cerebellum—lobule 6		L	212	5.16	−31	−63	−27
Cerebellum—lobule crus 1		L		4.97	−33	−59	−35
Cerebellum—lobule crus 1		L		4.69	−45	−67	−29
Superior parietal gyrus	7	L	143	5.11	−31	−61	58
Superior parietal gyrus	7	R	206	4.72	28	−61	50
Superior parietal gyrus	7	R		4.41	30	−63	58
Superior parietal gyrus	7	R		3.67	22	−67	62
*Mothers*_*child>adult*_ *> non‐mothers*_*child>adult*_							
Precuneus*	31	L	218	4.80	−1	−61	34
Precuneus*	31	L		4.15	−9	−63	36
Cuneus	7	L		3.60	−9	−69	28
*EXP*_*child>adult*_ *> IMP*_*child>adult*_							
Medial superior frontal gyrus*	9	L	1,357	9.53	−1	48	38
Superior frontal gyrus	8	R		7.76	16	38	54
Medial superior frontal gyrus*	8	L		5.84	−1	34	56
Caudate		R	132	5.86	6	16	10
Caudate		R		5.52	10	10	18
Caudate		R		4.98	12	22	10
*Non‐mothers*_*child>adult*_ *> mothers*_*child>adult*_	No clusters reached significance						
*IMP*_*child>adult*_ *> EXP*_*child>adult*_	No clusters reached significance						
*Interaction*	No clusters reached significance						

*Note*: Regions that showed significant differences in the same contrast in the hypotheses‐guided ROI analyses are indicated with an asterisk. All results are FWE‐corrected on the cluster level with *p* <.05. A 10% grey‐matter mask was used without performing small‐volume correction. Asterisks mark ROIs.

To sum up, both the ROI and the whole‐brain analyses supported Hypotheses 1, 2 and 3, but not Hypothesis 4.

## 4 DISCUSSION

This study used an implicit and explicit FAR task to investigate child‐evoked activation in women and modulations of child‐evoked activation due to motherhood and attention to affect. The affective child faces increased activity in face processing areas (bilateral fusiform and lingual gyri) and areas associated with social understanding (bilateral insulae, medial superior frontal gyrus). This child‐evoked activation was modulated by motherhood and attention to affect: First, mothers exhibited an increase of child‐evoked activation in the left precuneus. Second, child‐evoked activation was increased in the left medial superior frontal gyrus when participants were explicitly asked to recognise the displayed emotion instead of assessing an unrelated physical feature. There was no interaction of both modulating factors on child‐evoked activation. All results were independent of any behavioural differences between mothers and non‐mothers. However, response times in the implicit condition were more prolonged than in the explicit condition as well as longer in response to child compared to adult faces. In conclusion, affective child faces were processed preferentially compared to adult faces, and this child‐evoked activation was modulated by motherhood and increased by directing the attention to the affective content of the faces.

This study shows bilaterally stronger activation in both the core and the extended face processing system in response to affective child compared to adult faces in both mothers and non‐mothers, supporting Hypothesis 1. Concerning the extended system, there were effects both in areas associated with cognitive (medial superior frontal gyri) and affective (insulae) social understanding (see Figure 1). The reasons for this greater child‐evoked processing might be threefold: first, adults' faces are generally more similar to the faces of other adults than of children or infants (Lorenz, 1943). Therefore, child‐evoked activation could be due to the child faces being less similar to the participants' own adult faces. Second, it is possible that children's affective facial expression is less stereotypical and, therefore, harder to read, which demands larger neuronal activation. Third, child faces could lead to an increased emotional response compared to adult faces, which would explain why previous studies found increased task interference due to infant or child faces compared to adult faces (Thompson‐Booth et al., 2014b). Previous research also supports this by linking the right insula to reward and salience processing (Berridge & Kringelbach, 2015; Eckert et al., 2009; Phan et al., 2004; Vestergaard & Schultz, 2020). The child‐evoked activation in the right insula could indicate that child faces were more rewarding or salient for both mothers and non‐mothers. In addition to the ROI results, the explorative whole‐brain analysis revealed increased child‐evoked activation in the bilateral superior parietal gyri. These areas have been implicated in attentional processes, suggesting that child faces may have captured more attention (Kelley, Serences, Giesbrecht, & Yantis, 2008). To sum up, child‐evoked activation has been found both in the core and the extended system of our FAR model. This further cement the unique role children and their faces play in human society, indicating additional resources allocated to processing them compared to other adults.

Both the hypothesis‐guided ROI and the explorative whole‐brain analyses revealed modulation of child‐evoked activation by motherhood (Hypothesis 2), with mothers showing increased activation in the left precuneus of the extended system. The precuneus is a vital node in the default mode network (Fransson & Marrelec, 2008; Utevsky, Smith, & Huettel, 2014) and has been consistently associated with cognitive social understanding of emotions (Kogler et al., 2020; Schurz et al., 2020). Furthermore, studies have shown activation in the precuneus during interoception of emotions (Terasawa, Fukushima, & Umeda, 2013), inferring other people's emotions explicitly and implicitly in naturalistic settings (Wolf, Dziobek, & Heekeren, 2010) and reflecting on feelings (Ochsner et al., 2004). Therefore, precuneus activation could be associated with referencing oneself and retrieving self‐related memories—potentially concerning motherhood—as well as interoception, especially of affective states (Kogler et al., 2020; Northoff & Bermpohl, 2004). Thus, one possible explanation for mothers' increased child‐evoked activation in this area could be using different strategies compared to non‐mothers. Since mothers have a greater wealth of experiences with affective child faces than non‐mothers, who do not regularly interact with children, they might more strongly rely on their own experiences and emotions in reaction to the affective child faces. Additionally, training studies show that social understanding can be shaped by experience (Hildebrandt, McCall, & Singer, 2019; Trautwein, Kanske, Böckler, & Singer, 2020), and neural correlates of this effect have been linked to the precuneus (Rosenblau, O'Connell, Heekeren, & Dziobek, 2019). Since motherhood requires increased use of these processes, some of the differences between mothers and non‐mothers may be due to a training effect. Surprisingly, the precuneus was the only ROI that showed modulation of child‐evoked activation by motherhood. Neither face processing areas nor areas associated with affective social understanding showed modulation of child‐evoked activation by motherhood. This might indicate that mothers do not differ in their emotional response to these stimuli. However, future studies should investigate possible emotion‐specific modulations. To sum up, while child‐evoked activation was modulated by motherhood in the precuneus, an area associated with cognitive social understanding in the extended system, there was no modulation in areas of the core system or areas of the extended system associated with affective social understanding.

It is impossible to make claims about the direction of the modulatory effect of motherhood on child‐evoked activation based on the here presented data: while it may be the case that motherhood influences child‐evoked activation, it may as well be the case that having a brain system wired for preferential child processing more likely leads to motherhood. There are, however, several arguments to be made in support of the former statement, that is, a possible influence of motherhood on child‐evoked activation. First, longitudinal studies show structural and functional brain changes due to pregnancy and motherhood (Carmona et al., 2019; Hoekzema et al., 2017, 2020; Kim et al., 2010). Second, pregnancy and motherhood are associated with changes in female sex hormones, for example, estrogen and progesterone (Duarte‐Guterman, Leuner, & Galea, 2019; Kumar & Magon, 2012). These hormones have also been associated with emotion processing (Osório, Cassis, de Sousa, Poli‐Neto, & Martín‐Santos, 2018; Wu, Chen, et al., 2014; Wu, Zhou, et al., 2014). Therefore, changes in hormonal levels associated with pregnancy and motherhood could be a cause for modulations of child‐evoked activation. Third, differences between mothers and non‐mothers may be due to training effects as described above. However, there are also arguments for child‐evoked activation influencing motherhood, more precisely the likelihood of a woman becoming a mother. Although there was no difference between the importance that mothers and non‐mothers place on having a child, increased child‐evoked activation could nonetheless increase factors associated with the realisation of motherhood. For example, children may be more rewarding due to preferential child‐evoked activation, thereby increasing the motivation to have children. However, some of the emotions used in this study may not be associated with rewarding effects. While one may expect happy or neutral child faces to have a more rewarding effect on mothers, this may not hold for sad or fearful faces. Future research using only positive or emotionally neutral stimuli is needed to investigate this possibility. These factors may also interact with each other: increased preferential brain processing of child faces may increase the likelihood of becoming a mother, which in turn might further increase preferential brain processing of child faces. Apart from motherhood influencing child‐evoked activation and vice versa, it is also possible that factors are influencing *both* motherhood and child‐evoked activation. For example, non‐mothers may have used hormonal contraception more often and for a more extended period of their life. Studies indicate that hormonal contraception could alter the processing of affective faces (Hamstra, De Rover, De Rijk, & Van der Does, 2014; Marečková et al., 2014), therefore, the use of contraception could influence both child‐evoked activation and motherhood. Longitudinal studies on social understanding and motherhood are needed to disentangle these influences.

Although mothers and non‐mothers were comparable in most aspects tested in our sample, mothers were more likely to be in a relationship than non‐mothers. In combination with mothers and non‐mothers rating the importance of having children similarly, this could indicate that being in a relationship influenced the likelihood of an existing wish for children to be realised. Additionally, there may be other differences between mothers and non‐mothers that were not measured in this study. Some possible factors include sexual orientation and fertility. Wanting a child but not being able to have one may significantly influence how child faces are processed. Future studies should attempt to find subgroups of non‐mothers to specify whether the found differences generalise to all non‐mothers.

Concerning Hypothesis 3 on the modulation of child‐evoked activation by task, both ROI and whole‐brain analyses revealed stronger activation of the medial superior frontal gyrus in response to the explicit relative to the implicit task condition. The medial superior frontal gyrus is part of the extended face processing system and is associated with cognitive social understanding (see Figure 1). The explicit FAR task likely involves greater attention towards the displayed emotion. This could have led to increased reliance on processes of cognitive social understanding associated with the medial superior frontal gyrus (Schurz et al., 2020). Contrary to this, the implicit condition distracts the perceiver from the displayed emotion as it is irrelevant to the task. In this case, the difference between child and adult affect is decreased. There were no task‐related modulations of child‐evoked activation in any face processing areas or areas associated with affective social understanding. This might indicate that explicitly processing displayed emotions leads to more differences between affective child and adult faces in cognitive but not affective processes of social understanding. The modulation of child‐evoked activation by attention to affect did not interact with motherhood, therefore not supporting Hypothesis 4. This indicates independent modulation of child‐evoked activation by attention to affect and motherhood, specifically with attention to affect neither increasing nor decreasing the effect of motherhood. Both mothers and non‐mothers have increased child‐evoked activation in the medial superior frontal gyrus when attention is directed to the emotion in a child compared to an adult face.

All observed differences in neural processing between mothers and non‐mothers emerged despite no differences on the behavioural level. This suggests that mothers and non‐mothers did not differ in their performances as measured by response times in the explicit and implicit affect recognition task. There was no evidence for an interaction between motherhood and attention to affect or protagonist of the stimuli on the behavioural level. An explanation for missing behavioural effects may be that the task was chosen to increase detection efficiency for neural responses and not for sensitivity on the behavioural level. It is, therefore, possible that the task was not sensitive enough to detect behavioural differences as reported in other studies (Plank, Christiansen, et al., 2021; Proverbio et al., 2006). Additionally, it was harder to judge the unrelated physical features in the implicit condition than the displayed emotion in the explicit condition and make judgements about children than about adults in both the implicit and explicit condition for both mothers and non‐mothers alike. Even though there is only anecdotal evidence for the interaction between task and protagonist, the visual inspection of the data suggests that differences in response time between child and adult stimuli were more prominent in the implicit than in the explicit affect recognition task. This may be due to larger differences between children and adults in their physical features than their affective expressions. Further research using more sensitive behavioural measures is needed to investigate the interplay of motherhood, protagonist and FAR.

## 5 CONCLUSIONS

This study investigated child‐evoked activation during processing of facial affect and its modulation by motherhood and attention to affect. Affective child compared to adult faces led to increased activation in the core and the extended system of FAR in mothers and non‐mothers both during implicit and explicit affect recognition. Importantly, this child‐evoked activation was modulated by motherhood with stronger child‐evoked activation in the left precuneus in mothers compared to non‐mothers. This could indicate that mothers draw more heavily on the interoceptive investigation of their own emotions and personal memories when processing affective child faces. Additionally, the child‐evoked activation was increased in the medial superior frontal gyrus when participants were asked to recognise the displayed emotion compared to when they were asked to assess unrelated physical features. This suggests that differences between processing affective child and adult faces are increased when directing attention to the displayed emotion. There was no interaction between the modulatory effects of motherhood and attention to affect. This study is an essential contribution to studies showing differences in social understanding between mothers and non‐mothers, especially regarding the social understanding of children.

## CONFLICT OF INTEREST

The authors declare no potential conflict of interest.

## AUTHOR CONTRIBUTIONS

Irene Sophia Plank, Catherine Hindi Attar, Isabel Dziobek and Felix Bermpohl conceptualised and designed the study. Irene Sophia Plank took the lead on acquisition and analysis with vital contributions of Isabel Dziobek and Felix Bermpohl. Irene Sophia Plank wrote the draft. All authors contributed to the interpretation of the data and the revision of the manuscript. All authors provided critical feedback and helped shape the research, analysis and manuscript.

## Supporting information


**Appendix S1**: Supporting Information

## Data Availability

Data and scripts are available at https://osf.io/6qnps/ (Plank, Hindi Attar, et al., 2021).
